# Problematic Internet Use, Self-Esteem, and Well-Being Among Athletes: Testing a Mediation Model and Its Implications for Coaching

**DOI:** 10.3390/bs16081276

**Published:** 2026-07-27

**Authors:** Łukasz Bojkowski, Vivian de Oliveira, António Fernando Boleto Rosado

**Affiliations:** 1 Department of Sport Psychology, Poznan University of Physical Education, 61-871 Poznań, Poland; 2 Faculty of Human Kinetics, University of Lisbon, 1499-002 Lisbon, Portugal; vivian_oliveira58@hotmail.com (V.d.O.); arosado@fmh.ulisboa.pt (A.F.B.R.); 3 Interdisciplinary Center for the Study of Human Performance, 1499-002 Lisbon, Portugal

**Keywords:** internet addiction, internet use, mental health, self-esteem, well-being, sport psychology, coaching

## Abstract

The present study aimed to examine whether self-esteem acts as a mediator in the relationship between problematic internet use (PIU) and well-being among athletes, and to determine the implications of these relationships for educational practice, coaching strategies, and athlete support interventions. A total of 334 Polish athletes participated in the study and completed the Polish versions of the Generalized Problematic Internet Use Scale 2 (GPIUS2), the Rosenberg Self-Esteem Scale (SES), and the Mental Health Continuum-Short Form (MHC-SF). Pearson’s r correlation coefficients and mediation analyses using a bootstrap approach were conducted. The main results showed that PIU was negatively associated with self-esteem and well-being, whereas self-esteem was positively associated with well-being. Statistically significant, although small, indirect associations through self-esteem were observed between PIU and general well-being, as well as between all dimensions of PIU and emotional and psychological well-being. For social well-being, statistically significant indirect associations were observed only for preference for online social interaction and deficient self-regulation. These findings suggest that self-esteem may represent one psychological factor associated with the interrelations between PIU and well-being in athletes. The results may contribute to a more cautious understanding of the links between problematic internet use, self-esteem, and well-being, and may inform psychoeducational and preventive strategies in sport settings while acknowledging the limited practical significance of the observed effects.

## 1. Introduction

Problematic internet use has, in recent years, ceased to be a marginal phenomenon and has emerged as a global public health concern. Masaeli and Farhadi (2021), analyzing findings from numerous studies, indicated that during pandemic lockdowns, social isolation, stress, mood deterioration, and economic difficulties contributed to an increase in behaviors such as excessive use of the internet, smartphones, and online gaming. These activities began to serve as mechanisms for emotion regulation and escape from distress, thereby increasing the risk of developing compulsive patterns.

A review by Roberts et al. (2022) demonstrates that problematic internet use is associated with tangible psychological and social consequences. Individuals experiencing greater difficulties in this domain frequently exhibit comorbid symptoms and conditions, such as depression, anxiety, and other impairments in mental health and overall functioning. Similar conclusions were reported by Coco et al. (2025). Studies by Sahin Koybulan et al. (2024), Çelik and Haney (2023), and Bedir and Erhan (2021) further indicate that clinically significant levels of problematic internet use are also observed among athletic and physically active populations. This may be attributable to the specific context in which contemporary athletes operate, characterized by pressure, competition, and constant social exposure. Their daily functioning is increasingly intertwined with digital technologies—encompassing online communication, training monitoring, and social media engagement.

Scientific evidence indicates that problematic internet use is associated with reduced well-being. A study conducted by Stănculescu and Griffiths (2022) on a sample of 1202 participants demonstrated that individuals with high levels of internet addiction reported significantly lower levels of both hedonic well-being (happiness, life satisfaction) and eudaimonic well-being (sense of meaning, personal growth, effective functioning). Moreover, among individuals at high risk, characteristics such as preoccupation, mood modification, and impairments in social and occupational functioning were particularly pronounced. Similar conclusions were presented by Gong et al. (2024), who found that problematic internet use significantly increases levels of loneliness, which in turn contributes to a reduction in social and emotional well-being. Boer et al. (2020), analyzing data from 154,981 adolescents across 29 countries, demonstrated that problematic social media use is negatively associated with multiple dimensions of well-being, including mental (life satisfaction, psychological complaints), school-related (school satisfaction, school pressure), and social (family support, peer support) domains, with only problematic—not intensive—use being consistently linked to poorer functioning.

Research findings also suggest that problematic internet use may serve a compensatory function in response to reduced well-being. However, in the long term, it may reinforce maladaptive coping strategies, particularly in the domains of emotion regulation and interpersonal relationships (Yildirim & Caner-Yildirim, 2025). Malas et al. (2025) emphasize that problematic internet use should not be viewed as an isolated phenomenon, but rather as part of a broader lifestyle pattern associated with quality of life and mental well-being. This perspective is further supported by findings from studies involving physically active populations (Marazziti et al., 2023; Piepiora et al., 2024). Within athletic populations, this issue may be particularly relevant because psychological well-being represents a key prerequisite for optimal sports performance. Consequently, factors that undermine well-being, such as problematic internet use, may adversely affect athletic functioning and performance outcomes (Fadare et al., 2022).

As indicated by previous research, problematic internet use may be significantly associated with the well-being of individuals across different age groups, including those who are physically active. In our study, it was assumed that this relationship is mediated by the level of self-esteem. The rationale for this model is supported, among others, by the findings of Barry et al. (2024), conducted on a sample of 1120 student-athletes, which demonstrated that problematic social media use was linked to both lower self-esteem and reduced well-being. This suggests that not only the amount of use, but primarily dysfunctional patterns of engagement, may negatively affect self-perception. Similar relationships were confirmed by a meta-analysis by Huang (2022), encompassing 133 samples (N = 244,676), which showed that problematic social media use was negatively related to self-esteem and life satisfaction, and positively associated with depression and loneliness. Likewise, Mousoulidou et al. (2024) reported that both internet addiction and social media addiction were negatively associated with self-esteem and positively associated with symptoms of depression, anxiety, and stress. Additional support for the proposed mediation model is provided by evidence suggesting that problematic internet use may undermine individuals’ self-perceptions and psychological functioning, thereby indirectly affecting overall well-being. For example, Lin et al. (2023) demonstrated that greater time spent online was associated with higher levels of problematic internet use. Taken together, these findings suggest that self-esteem may represent an important psychological mechanism linking problematic internet use and well-being.

The aim of the present study is therefore to examine whether self-esteem acts as a potential mediator in the relationship between problematic internet use and well-being among athletes. The proposed direction of the mediation model assumes that problematic internet use is associated with self-esteem, and that self-esteem is subsequently associated with well-being. This direction was selected on the basis of prior theoretical and empirical work suggesting that problematic internet use may be linked to lower self-esteem, which in turn is related to poorer psychological well-being. At the same time, given evidence indicating a bidirectional relationship between self-esteem and problematic internet use (Lin et al., 2023; Lai et al., 2023), this model should be interpreted as theoretically grounded rather than causal, and alternative directions of association cannot be excluded.

Considering the impact of problematic internet use on athletes’ well-being, as evidenced by the literature presented above, it is important to discuss the role of sports coaches, teachers, mentors, and consultants. Their work in contemporary sport is no longer limited to performance enhancement but increasingly involves a holistic approach that includes supporting psychological development, facilitating life-skill acquisition, managing groups, and organizing athletes’ daily functioning. At the same time, it is widely recognized that such professionals influence athletes’ self-esteem, sense of self-worth, and, consequently, their overall well-being (Granero-Gallegos et al., 2023; Coussens et al., 2025). In addition, coaches, teachers, and consultants may increasingly serve as digital educators by supporting the organization of daily routines, guiding the use of digital tools, and explaining the benefits and risks associated with digital environments (Gould et al., 2020; Geurin, 2023). Therefore, a better understanding of the relationships among problematic internet use, self-esteem, and well-being may have valuable implications for educational and coaching practice and contribute to the development of more effective strategies for supporting athletes’ psychological functioning and long-term development.

Against the background of existing research, this project offers two important contributions to the literature. First, it focuses exclusively on athletic populations, which remain relatively underexplored with regard to the relationships among problematic internet use, self-esteem, and well-being. Second, in contrast to many previous studies that have relied primarily on correlational approaches, this study directly tests a mediation model. Furthermore, identifying self-esteem as a potential mechanism may provide implications for educational and coaching interventions aimed at promoting athletes’ psychological well-being and long-term development.

## 2. Method

### 2.1. Participants

A total of 334 athletes aged 19 to 30 years (M = 21.86; SD = 2.14) participated in the study, including 147 women (44%) and 187 men (56%). The training experience of all participants ranged from 3 to 19 years (M = 9.81; SD = 4.01). Participants were recruited using a convenience sampling strategy, which should be taken into account when interpreting the generalizability of the findings. The study sample included individuals training in various sports disciplines, such as acrobatics, badminton, athletics (sprint running, long-distance running, pole vault), Brazilian jiu-jitsu, gymnastics, field hockey, equestrian sports, judo, canoeing, basketball, lacrosse, MMA, cycling, football, handball, swimming, weightlifting, volleyball, fencing, dance, table tennis, tennis, strength training, triathlon, powerlifting, rowing, sport climbing, wrestling, and sailing. Among the participants, 160 individuals (48%) were students at Poznan University of Physical Education (Poznań, Poland). Furthermore, 51% of participants reported competing at the regional level, 36% at the national level, and 13% at the international level. All participants declared Polish citizenship. Although the sample included athletes from various sports and competitive levels, its homogeneity in nationality, age, and partial institutional affiliation limits generalizability. No a priori power analysis was conducted, and the sample size resulted from the availability of eligible participants.

The study was conducted in March and April 2026. The research protocol was submitted to the Bioethics Committee of the Poznan University of Medical Sciences (Poznań, Poland), which issued a statement indicating that the study did not require formal ethical approval (decision dated 12 March 2026).

Participation in the study was voluntary, and participants could withdraw at any time without providing a reason. The study was anonymous, and the data were analyzed exclusively in aggregate form, without the possibility of identifying individual participants. Data collection was conducted in person on non-training days by a researcher who is a licensed psychologist. Participants received no financial compensation or other incentives for their participation. All procedures were conducted in accordance with the ethical principles of the Declaration of Helsinki, and participants were informed in detail about the study procedures prior to data collection.

The inclusion criteria were: adulthood (>18 years), possession of a valid athlete license or membership in a sports club, engaging in training at least three times per week, participation in sports competitions (of any level) within the last twelve months, and no injuries that could hinder participation in the study.

### 2.2. Research Tools

The Generalized Problematic Internet Use Scale 2 (GPIUS2)—in the Polish adaptation by Biechowska et al. (2012)—is a tool based on Caplan’s conceptual framework, designed to assess the severity of problematic internet use and the risk of developing behavioral addictions related to online activity. It should be noted that the Polish adaptation used in the present study is based on unpublished material.

The instrument consists of 15 items rated on a seven-point Likert scale, with the total score ranging from 15 to 105 points, allowing for classification of risk levels. The content of the items reflects general problematic internet use (GPIU) as well as four specific dimensions: preference for online social interaction, mood regulation, negative outcomes, and deficient self-regulation (Biechowska et al., 2012). The Polish adaptation has been described by its authors as demonstrating satisfactory psychometric properties. Accordingly, this should be considered when interpreting the results obtained with the GPIUS2 in the present sample. In the broader context of behavioral addiction research in Poland, it has been shown that similar instruments (e.g., for measuring gaming disorder) achieve high levels of reliability and construct validity, which confirms the adequacy of measurement methods for this type of phenomenon (Cudo et al., 2024). Nevertheless, further studies are needed to provide more comprehensive validation evidence for the Polish version of the GPIUS2, including its factor structure and reliability in Polish samples.

The Rosenberg Self-Esteem Scale (SES)—in the Polish adaptation by Dzwonkowska et al. (2007)—is a classic instrument used to measure global self-esteem, understood as an overall attitude toward oneself.

The scale consists of 10 items rated on a Likert scale and is characterized by a unidimensional structure. The Polish adaptation demonstrates high reliability (Cronbach’s α in the range of 0.81–0.83) as well as good theoretical validity, confirming its usefulness in both scientific research and diagnostic practice (Dzwonkowska et al., 2007). This scale is widely used as an indicator of psychological functioning and is often employed in the validation of other instruments, showing significant associations with psychological well-being, life satisfaction, and psychopathological symptoms (Kocur et al., 2025).

The Mental Health Continuum-Short Form (MHC-SF)—in the Polish adaptation by Karaś et al. (2014)—is a widely used instrument for assessing the level of psychological well-being, understood as positive functioning of an individual in emotional, psychological, and social domains.

The questionnaire consists of 14 items referring to the frequency of experiencing various well-being states over the past month, rated on a six-point frequency scale. The instrument operationalizes well-being as one general well-being as well as three specific dimensions, including emotional well-being (e.g., feelings of happiness and life satisfaction), psychological well-being (e.g., personal growth, autonomy, purpose in life), and social well-being (e.g., sense of belonging and perceived meaningfulness of society) (Karaś et al., 2014). The Polish adaptation demonstrates very good psychometric properties—its three-factor structure, high reliability, and construct validity have been confirmed. Studies conducted in the Polish population indicate that MHC-SF scores are significantly associated with the level of social support and the quality of interpersonal relationships (Adamczyk & Segrin, 2015).

### 2.3. Statistical Analysis

To assess the strength and direction of relationships among all examined variables (general problematic internet use and its four dimensions, self-esteem, general well-being and its three dimensions), Pearson’s r correlation coefficient was used. To examine the mediating role of self-esteem in the relationship between manifestations of problematic internet use and the well-being experienced by participants, a mediation analysis was conducted. In this analysis, general problematic internet use (and its dimensions) was treated as an independent variable, general well-being (and its dimensions) as a dependent variable, and self-esteem as the mediator.

The mediation effect was considered significant when the indirect effect of the independent variable on the dependent variable—through the mediator—was statistically significant. The significance of mediation effects was tested using the percentile bootstrap method (5000 samples). The effect was considered significant when the 95% confidence interval did not include zero (Hayes, 2017). Statistical analyses were conducted using Jamovi software (version 2.6.26).

Additionally, common method variance (CMV) was assessed using Harman’s single-factor test (Podsakoff et al., 2003). The analysis examined the percentage of variance in all items explained by the first unrotated factor. The results showed that this factor accounted for 26% of the total variance in the test items. This value is below the commonly accepted threshold of 50%, suggesting that the analyzed data are not substantially affected by common method bias (Podsakoff et al., 2003).

## 3. Results

In the first stage, Pearson’s r correlation analysis was conducted between all analyzed variables. Subsequently, a mediation analysis was performed in which general problematic internet use was treated as the independent variable and general well-being as the dependent variable. In the following stages, analyses were conducted considering the individual dimensions of both scales.

All four dimensions of general problematic internet use (preference for online social interaction, mood regulation, negative outcomes, and deficient self-regulation) were positively correlated with each other (r = 0.484–0.667; *p* < 0.001), as well as with the overall score (r = 0.742–0.903; *p* < 0.001). Similarly, all three dimensions of general well-being (emotional, psychological, and social well-being) were positively correlated with each other (r = 0.626–0.722; *p* < 0.001), as well as with the corresponding overall score (r = 0.838–0.910; *p* < 0.001).

General problematic internet use was negatively correlated with general well-being (r = −0.250; *p* < 0.001) and all of its dimensions (r = −0.151 to −0.319; *p* < 0.01–0.001). These associations were statistically significant but generally small in magnitude. Preference for online social interaction was negatively correlated with general well-being (r = −0.160; *p* < 0.01), as well as with two of its dimensions—emotional (r = −0.159; *p* < 0.01) and social well-being (r = −0.199; *p* < 0.001). Mood regulation was negatively correlated with general well-being (r = −0.256; *p* < 0.001) and all three of its dimensions (r = −0.157 to −0.289; *p* < 0.01–0.001). Negative outcomes showed a negative correlation with general well-being (r = −0.250; *p* < 0.001) and all three of its dimensions (r = −0.166 to −0.299; *p* < 0.01–0.001). In turn, deficient self-regulation was negatively correlated with general well-being (r = −0.182; *p* < 0.001) and two dimensions—emotional (r = −0.158; *p* < 0.01) and social well-being (r = −0.270; *p* < 0.001).

Self-esteem was negatively correlated with general problematic internet use (r = −0.219; *p* < 0.001) and its four dimensions (r = −0.161 to −0.209; *p* < 0.01–0.001), while being positively correlated with general well-being (r = 0.201; *p* < 0.001) and its three dimensions (r = 0.176–0.183; *p* < 0.01–0.001). These correlations were statistically significant but small, suggesting that the relationships among self-esteem, problematic internet use, and well-being should be interpreted with caution in terms of their practical significance (Table 1).

A statistically significant, although small, indirect association was observed between general problematic internet use and general well-being through self-esteem (b = −0.0327; 95% C.I. [−0.0693; −0.0067]; β = −0.0336). Thus, self-esteem may be considered a modest potential mediator in this relationship.

General problematic internet use was negatively associated with self-esteem (b = −0.0416; 95% C.I. [−0.0632; −0.0198]; β = −0.2190). In turn, self-esteem was positively associated with general well-being (b = 0.7871; 95% C.I. [0.2298; 1.3326]; β = 0.1534) (Table 2).

Statistically significant, although small, indirect associations through self-esteem were observed between preference for online social interaction (b = −0.0250; 95% C.I. [−0.0550; −0.0042]; β = −0.0258), mood regulation (b = −0.0246; 95% C.I. [−0.0554; −0.0043]; β = −0.0249), negative outcomes (b = −0.0281; 95% C.I. [−0.0589; −0.0060]; β = −0.0309), and deficient self-regulation (b = −0.0163; 95% C.I. [−0.0362; −0.0032]; β = −0.0284) and emotional well-being through self-esteem. These findings suggest that self-esteem may represent a modest potential mediator in these relationships.

Preference for online social interaction (b = −0.1253; 95% C.I. [−0.2094; −0.0404]; β = −0.1675), mood regulation (b = −0.1224; 95% C.I. [−0.2052; −0.0389]; β = −0.1609), negative outcomes (b = −0.1464; 95% C.I. [−0.2243; −0.0698]; β = −0.2086), and deficient self-regulation (b = −0.0829; 95% C.I. [−0.1351; −0.0337]; β = −0.1869) were negatively associated with self-esteem, which in turn was positively associated with emotional well-being (b = 0.1995; 95% C.I. [0.0689; 0.3306]; β = 0.1539) (Table 3).

Statistically significant, although small, indirect associations through self-esteem were observed between preference for online social interaction (b = −0.0511; 95% C.I. [−0.1100; −0.0091]; β = −0.0291), mood regulation (b = −0.0438; 95% C.I. [−0.0958; −0.0071]; β = −0.0246), negative outcomes (b = −0.0519; 95% C.I. [−0.1092; −0.0099]; β = −0.0315), and deficient self-regulation (b = −0.0343; 95% C.I. [−0.0710; −0.0080]; β = −0.0330) and psychological well-being through self-esteem. These findings suggest that self-esteem may represent a modest potential mediator in these relationships.

Preference for online social interaction (b = −0.1253; 95% C.I. [−0.2120; −0.0400]; β = −0.1675), mood regulation (b = −0.1224; 95% C.I. [−0.2061; −0.0413]; β = −0.1609), negative outcomes (b = −0.1464; 95% C.I. [−0.2234; −0.0696]; β = −0.2086), and deficient self-regulation (b = −0.0829; 95% C.I. [−0.1347; −0.0341]; β = −0.1869) were negatively associated with self-esteem, which in turn was positively associated with psychological well-being (b = 0.4080; 95% C.I. [0.1500; 0.6509]; β = 0.1738) (Table 4).

Statistically significant, although small, indirect associations through self-esteem were observed between preference for online social interaction (b = −0.0390; 95% C.I. [−0.0901; −0.0056]; β = −0.0245) and deficient self-regulation (b = −0.0228; 95% C.I. [−0.0541; −0.0030]; β = −0.0243) and social well-being through self-esteem.

In the remaining two cases, no statistically significant indirect associations were observed. Given the small standardized indirect effects, these findings should be interpreted with caution, particularly in terms of their practical significance.

Preference for online social interaction (b = −0.1253; 95% C.I. [−0.2128; −0.0386]; β = −0.1675), mood regulation (b = −0.1224; 95% C.I. [−0.2044; −0.0377]; β = −0.1609), negative outcomes (b = −0.1464; 95% C.I. [−0.2197; −0.0712]; β = −0.2086), and deficient self-regulation (b = −0.0829; 95% C.I. [−0.1354; −0.0339]; β = −0.1869) were negatively associated with self-esteem, which in turn was positively associated with social well-being (b = 0.3108; 95% C.I. [0.0888; 0.5298]; β = 0.1464) (Table 5).

## 4. Discussion

Problematic internet use has become a global public health concern, particularly in the context of increased stress, isolation, and emotional difficulties, which may be associated with individuals’ greater reliance on online activities as coping strategies (Masaeli & Farhadi, 2021). Research consistently shows that excessive and dysfunctional internet use is associated with negative psychological, social, and physical outcomes (Roberts et al., 2022; Coco et al., 2025). This issue is also evident among physically active individuals and athletes, whose functioning is increasingly intertwined with digital environments (Sahin Koybulan et al., 2024; Çelik & Haney, 2023; Bedir & Erhan, 2021). Empirical evidence indicates that problematic internet use is linked to reduced well-being, both in its hedonic and eudaimonic dimensions (Stănculescu & Griffiths, 2022), as well as increased loneliness and poorer social functioning (Gong et al., 2024; Boer et al., 2020). Importantly, prior studies suggest that this relationship may be partly associated with underlying psychological mechanisms, particularly self-esteem. Lower self-esteem has been associated with higher levels of problematic internet use and poorer well-being (Huang, 2022; Mousoulidou et al., 2024). Therefore, the present study aims to examine whether self-esteem may act as a potential mediator in the relationship between problematic internet use and well-being among athletes. Given the cross-sectional design of the study, this model is interpreted in terms of associations rather than causal effects.

The results of the present study showed that all dimensions of general problematic internet use were negatively associated with self-esteem, whereas self-esteem was positively related to all dimensions of well-being. Regarding the mediation model, statistically significant, although small, indirect associations through self-esteem were found between problematic internet use and both overall well-being and most of its specific dimensions. For social well-being, two indirect associations did not reach the conventional threshold of statistical significance. However, these effects were marginally non-significant and similar in magnitude to those that were statistically significant. Overall, the findings are consistent with previous research indicating that well-being and self-esteem are associated with problematic internet use, with higher levels of problematic use generally linked to poorer indicators in both domains (Barry et al., 2024; Mei et al., 2016).

With regard to self-esteem, there is extensive literature supporting its bidirectional association with problematic internet use (Lin et al., 2023; Wacks & Weinstein, 2021; Lai et al., 2023). For example, Lee and Kim (2018) identified low self-esteem as one of the primary factors associated with excessive smartphone use among adolescents. One possible explanation for this relationship is that individuals with low self-esteem may turn to online environments as a coping strategy when dealing with personal difficulties (Koronczai et al., 2013), with problematic internet use potentially functioning as a compensatory mechanism in individuals experiencing lower levels of well-being (Yildirim & Caner-Yildirim, 2025). The use of the internet for this purpose can be interpreted in light of the Compensatory Internet Use Theory (Kardefelt-Winther, 2014), which posits that individuals may resort to online environments to fulfill unmet needs in their everyday lives and to cope with negative emotions. In addition, individuals with low self-esteem may have difficulties controlling behaviors related to problematic internet use, experience discrepancies between their real and ideal self, and show lower awareness of the potential negative consequences of such use (Mei et al., 2016). These potential processes may be particularly relevant in athletic populations, as low self-esteem is consistently associated with poorer mental health outcomes (Walton et al., 2021), whereas higher self-esteem has been recognized as a factor that may be associated with confidence, resilience, and performance in competitive sport (Stoyanova & Ivantchev, 2025).

Similarly, previous studies have demonstrated associations between problematic internet use and well-being (Đurić et al., 2024; Gong et al., 2024; Stănculescu & Griffiths, 2022), with the former being negatively related to the latter, both in the psychological domain and in the physical and social domains (Cai et al., 2023; Coco et al., 2025). On the other hand, individuals with higher levels of well-being tend to show a lower propensity for problematic internet-related behaviors (Marino et al., 2018), whereas lower levels of well-being have been identified as a correlate or potential risk factor associated with problematic internet use (Đurić et al., 2024), suggesting that well-being may function as one of several factors linked to lower vulnerability to this phenomenon.

Another potential protective factor, not examined in the present study but emphasized in the literature, is physical activity itself. Research indicates a negative relationship between smartphone and internet use and engagement in physical activity (Coco et al., 2025). However, the relationship within athletic populations may be more complex and should be interpreted within the specific context of competitive sport. In contrast to recreational physical activity, competitive sport often involves high training demands, performance pressure, intensive competition, and a stressful lifestyle, all of which may be associated with athletes’ mental health (Schinke et al., 2017). These conditions may, in turn, be linked to greater vulnerability to maladaptive or addictive behaviors, including problematic internet use (Sahin Koybulan et al., 2024), which has been associated with adverse outcomes such as depressive symptoms among athletes (Çelik & Haney, 2023).

In light of the above, the present study examined self-esteem as a potential mediator in the relationship between problematic internet use and well-being. This approach and results are consistent with previous research, particularly among adolescent populations. Lin et al. (2023) demonstrated that self-esteem may play a mediating role in the relationship between problematic internet use and subjective well-being. Lai et al. (2023), in a longitudinal study, examined the relationships between problematic internet use, self-esteem, and depressive symptoms, indicating their stability over time. Specifically, higher levels of problematic internet use were associated with lower self-esteem, and both factors were linked to subsequent increases in depressive symptoms. However, given the cross-sectional design of the present study and the small magnitude of the observed indirect effects, the mediating role of self-esteem should be interpreted cautiously and in terms of statistical association rather than causal explanation.

However, the lack of studies examining this mediation in athletic populations limits the possibilities for comparison and interpretation of the present findings. To the best of our knowledge, this is among the first studies to investigate these variables and their interrelationships among athletes. Considering that athletes’ self-esteem may differ in nature from that of non-athletes (Petrovska et al., 2022), the specific characteristics of the sports context may be relevant to the observed relationships. In competitive sport, self-esteem is often strongly linked to performance outcomes, coach evaluations, social exposure, and interpersonal comparisons, which may make it more dynamic and dependent on sport-related experiences. Nevertheless, the modest effect sizes observed in the present study suggest that self-esteem should be regarded as one of several possible factors associated with athletes’ well-being, rather than as a dominant explanatory mechanism.

At the same time, the findings of the present study suggest that the potential mediating role of self-esteem was broadly similar across the different dimensions of well-being, although some indirect associations differed with respect to whether they crossed the conventional threshold for statistical significance. Thus, rather than treating these differences as clear evidence that self-esteem operates differently in relation to social well-being, they should be interpreted cautiously as preliminary patterns that require further verification. It is possible that social well-being among athletes is more strongly shaped by mechanisms related to interpersonal relationships and group functioning. This interpretation is broadly consistent with the findings of Bedir and Erhan (2021), who showed that the variables included in their model explained more than 60% of the variance in problematic internet use among athletes, highlighting the potential importance of psychosocial factors in this population.

## 5. Practical Implications for Coaching

The findings of the present study also have potential practical implications for the sports context. Since self-esteem appears to play a modest but statistically significant mediating role in the relationship between problematic internet use and well-being, interventions aimed at promoting athletes’ mental health may consider strengthening self-esteem as one of several potential protective factors. Thus, the results should not be understood as evidence that coaching interventions focused on self-esteem will directly or substantially improve athletes’ well-being, but rather as indicating one possible area for supportive practice. Given that coaches, teachers, consultants, and mentors may be associated with the development of athletes’ self-esteem (Granero-Gallegos et al., 2023; Coussens et al., 2025), their educational and coaching practices could incorporate strategies aimed at supporting this aspect of psychological functioning as part of developmental support.

In addition, coaches, teachers, sport psychologists, consultants, mentors, and other professionals working with athletes may benefit from implementing psychoeducational and preventive strategies that monitor problematic patterns of internet use. Such interventions may contribute to addressing the potential negative consequences associated with maladaptive online behaviors, including lower levels of well-being. At the same time, professionals working with athletes may play a supportive role in their digital education by increasing awareness of both the risks and benefits associated with digital technologies and online environments (Gould et al., 2020; Geurin, 2023). Fostering digital competencies and promoting healthy technology-related habits may encourage more balanced patterns of internet use and may support athletes’ well-being. Achieving these goals, however, requires that professionals themselves receive appropriate preparation, develop relevant competencies, and systematically update their knowledge in line with current developments in this area.

## 6. Limitations and Future Recommendations

Several limitations of the present study should be acknowledged. First, gender differences were not considered in the analyses. The absence of such an analysis limits the ability to draw more precise conclusions regarding the nature of the relationships under investigation. Second, no distinction was made with respect to the type of sport practiced. It can be assumed that athletes engaged in team sports, due to greater social support and more frequent interpersonal interactions, may experience different relationships between problematic internet use, self-esteem, and well-being compared to individual athletes, who more often function in relatively isolated conditions. Third, the study employed a cross-sectional design, which precludes causal inferences. Although a mediation model was tested, it is not possible to determine the directionality of the relationships between the examined variables. Therefore, the mediation results should be interpreted as indirect statistical associations rather than evidence of causal mechanisms. Moreover, the observed correlations and standardized indirect effects were small in magnitude, which further limits the strength and practical scope of the conclusions. In addition, no a priori sample size analysis was conducted using G*Power, which limits the ability to determine whether the obtained sample size was sufficient to detect the expected effects. Another limitation is that no correction for multiple comparisons was applied, despite the testing of several mediation models. Consequently, some statistically significant findings, particularly those with p-values close to the significance threshold and narrow bootstrap confidence intervals, should be interpreted as preliminary and with caution. An additional limitation concerns the use of the Polish version of the GPIUS2 based on unpublished adaptation material. Finally, only self-report measures were used, which entails the risk of biases such as response bias, social desirability effects, and inaccuracies in assessing one’s own behaviors and psychological states.

Future research should consider incorporating analyses of gender differences and comparing athletes representing different types of sports (team vs. individual). It is also recommended to conduct longitudinal studies, which would enable the verification of the directionality of these relationships and allow for more accurate testing of mediation models. Another important step would be the use of more diverse measurement methods, such as behavioral data (e.g., actual time spent using the internet), coach assessments, or physiological indicators, which could help reduce the impact of self-report biases. Future studies should also include correction procedures for multiple comparisons or other strategies for controlling the risk of false-positive findings, particularly when testing numerous mediation models. Furthermore, it would be valuable to extend the model by including additional psychological variables, such as emotion regulation, social comparison, or social support, which may act as mediators or moderators in the relationship between problematic internet use and well-being. Such extensions may help determine whether self-esteem has a unique practical role or whether it should be understood as one component within a broader network of psychological and social factors associated with athletes’ well-being. Finally, future intervention-based research would be needed before stronger coaching recommendations can be formulated.

## Figures and Tables

**Table 1:** Pearson’s r correlation coefficients—General problematic internet use and its four dimensions, and self-esteem, as well as general well-being and its three dimensions.

Variables	Correlations N = 334								
	POSI	MR	NO	DSF	GenPIU	EWB	PWB	SWB	GenWB
**POSI**	—								
**MR**	0.484 ***	—							
**NO**	0.507 ***	0.594 ***	—						
**DSF**	0.539 ***	0.667 ***	0.637 ***	—					
**GenPIU**	0.742 ***	0.818 ***	0.820 ***	0.903 ***	—				
**EWB**	−0.159 **	−0.157 **	−0.166 **	−0.158 **	−0.192 ***	—			
**PWB**	−0.082	−0.211 ***	−0.183 ***	−0.066	−0.151 **	0.626 ***	—		
**SWB**	−0.199 ***	−0.289 ***	−0.299 ***	−0.270 ***	−0.319 ***	0.722 ***	0.686 ***	—	
**GenWB**	−0.160 **	−0.256 ***	−0.250 ***	−0.182 ***	−0.250 ***	0.838 ***	0.900 ***	0.910 ***	—
**SE**	−0.168 **	−0.161 **	−0.209 ***	−0.187 ***	−0.219 ***	0.176 **	0.183 ***	0.176 **	0.201 ***

*Note:* POSI—Preference for online social interaction; MR—Mood regulation; NO—Negative outcomes; DSF—Deficient self-regulation; GenPIU—General problematic internet use (overall score); EWB—Emotional well-being; PWB—Psychological well-being; SWB—Social well-being; GenWB—General well-being (overall score); SE—Self-esteem. * *p* < 0.05; ** *p* < 0.01; *** *p* < 0.001.

**Table 2:** Self-esteem as a mediator in the relationship between general problematic internet use and general well-being—Results of the mediation analysis.

Mediator Models N = 334								
General problematic internet use (overall score) ⇒ Self-esteem ⇒ General well-being (overall score)								
				**95% C.I.**				
**Type**	**Effect**	**Estimate**	**SE**	**Lower**	**Upper**	**β**	**z**	***p***
**Indirect**	GenPIU ⇒ SE ⇒ GenWB	−0.0327	0.0139	−0.0693	−0.0067	−0.0336	−2.35	0.019
**Component**	GenPIU ⇒ SE	−0.0416	0.0101	−0.0632	−0.0198	−0.2190	−4.10	<0.001
	SE ⇒ GenWB	0.7871	0.2753	0.2298	1.3326	0.1534	2.86	0.004
**Direct**	GenPIU ⇒ GenWB	−0.2105	0.0522	−0.3331	−0.0852	−0.2162	−4.03	<0.001
**Total**	GenPIU ⇒ GenWB	−0.2432	0.0517	−0.3634	−0.1247	−0.2498	−4.71	<0.001

*Note:* GenPIU—General problematic internet use (overall score); SE—Self-esteem; GenWB—General well-being (overall score). Confidence intervals computed with method: Bootstrap percentiles 5000 (interval 95%); betas are completely standardized effect size.

**Table 3:** Results of the mediation analysis—Self-esteem as a mediator in the relationship between the four dimensions of general problematic internet use (preference for online social interaction, mood regulation, negative outcomes, deficient self-regulation) and emotional well-being (a dimension of general well-being).

Mediator Models N = 334								
Preference for online social interaction ⇒ Self-esteem ⇒ Emotional well-being Mood regulation ⇒ Self-esteem ⇒ Emotional well-being Negative outcomes ⇒ Self-esteem ⇒ Emotional well-being Deficient self-regulation ⇒ Self-esteem ⇒ Emotional well-being								
				**95% C.I.**				
**Type**	**Effect**	**Estimate**	**SE**	**Lower**	**Upper**	**β**	**z**	***p***
**Indirect**	POSI ⇒ SE ⇒ EWB	−0.0250	0.0119	−0.0550	−0.0042	−0.0258	−2.10	0.036
	MR ⇒ SE ⇒ EWB	−0.0246	0.0119	−0.0554	−0.0043	−0.0249	−2.06	0.039
	NO ⇒ SE ⇒ EWB	−0.0281	0.0126	−0.0589	−0.0060	−0.0309	−2.23	0.026
	DSF ⇒ SE ⇒ EWB	−0.0163	0.0075	−0.0362	−0.0032	−0.0284	−2.18	0.029
**Component**	POSI ⇒ SE	−0.1253	0.0404	−0.2094	−0.0404	−0.1675	−3.11	0.002
	MR ⇒ SE	−0.1224	0.0411	−0.2052	−0.0389	−0.1609	−2.98	0.003
	NO ⇒ SE	−0.1464	0.0376	−0.2243	−0.0698	−0.2086	−3.90	<0.001
	DSF ⇒ SE	−0.0829	0.0238	−0.1351	−0.0337	−0.1869	−3.48	<0.001
	SE ⇒ EWB	0.1995	0.0702	0.0689	0.3306	0.1539	2.84	0.004
**Direct**	POSI ⇒ EWB	−0.1288	0.0525	−0.2502	−0.0127	−0.1328	−2.45	0.014
	MR ⇒ EWB	−0.1307	0.0533	−0.2479	−0.0176	−0.1325	−2.45	0.014
	NO ⇒ EWB	−0.1232	0.0496	−0.2336	−0.0078	−0.1354	−2.48	0.013
	DSF ⇒ EWB	−0.0746	0.0313	−0.1443	−0.0054	−0.1297	−2.39	0.017
**Total**	POSI ⇒ EWB	−0.1538	0.0525	−0.2768	−0.0326	−0.1586	−2.93	0.003
	MR ⇒ EWB	−0.1552	0.0534	−0.2712	−0.0463	−0.1574	−2.91	0.004
	NO ⇒ EWB	−0.1513	0.0492	−0.2601	−0.0468	−0.1663	−3.08	0.002
	DSF ⇒ EWB	−0.0909	0.0311	−0.1607	−0.0269	−0.1581	−2.92	0.003

*Note:* POSI—Preference for online social interaction; MR—Mood regulation; NO—Negative outcomes; DSF—Deficient self-regulation; SE—Self-esteem; EWB—Emotional well-being. Confidence intervals computed with method: Bootstrap percentiles 5000 (interval 95%); betas are completely standardized effect size.

**Table 4:** Results of the mediation analysis—Self-esteem as a mediator in the relationship between the four dimensions of general problematic internet use (preference for online social interaction, mood regulation, negative outcomes, deficient self-regulation) and psychological well-being (a dimension of general well-being).

Mediator Models N = 334								
Preference for online social interaction ⇒ Self-esteem ⇒ Psychological well-being Mood regulation ⇒ Self-esteem ⇒ Psychological well-being Negative outcomes ⇒ Self-esteem ⇒ Psychological well-being Deficient self-regulation ⇒ Self-esteem ⇒ Psychological well-being								
				**95% C.I.**				
**Type**	**Effect**	**Estimate**	**SE**	**Lower**	**Upper**	**β**	**z**	***p***
**Indirect**	POSI ⇒ SE ⇒ PWB	−0.0511	0.0230	−0.1100	−0.0091	−0.0291	−2.23	0.026
	MR ⇒ SE ⇒ PWB	−0.0438	0.0213	−0.0958	−0.0071	−0.0246	−2.06	0.039
	NO ⇒ SE ⇒ PWB	−0.0519	0.0229	−0.1092	−0.0099	−0.0315	−2.26	0.024
	DSF ⇒ SE ⇒ PWB	−0.0343	0.0145	−0.0710	−0.0080	−0.0330	−2.36	0.018
**Component**	POSI ⇒ SE	−0.1253	0.0404	−0.2120	−0.0400	−0.1675	−3.11	0.002
	MR ⇒ SE	−0.1224	0.0411	−0.2061	−0.0413	−0.1609	−2.98	0.003
	NO ⇒ SE	−0.1464	0.0376	−0.2234	−0.0696	−0.2086	−3.90	<0.001
	DSF ⇒ SE	−0.0829	0.0238	−0.1347	−0.0341	−0.1869	−3.48	<0.001
	SE ⇒ PWB	0.4080	0.1279	0.1500	0.6509	0.1738	3.19	0.001
**Direct**	POSI ⇒ PWB	−0.0924	0.0957	−0.3010	0.1021	−0.0526	−0.97	0.334
	MR ⇒ PWB	−0.3334	0.0956	−0.5422	−0.1153	−0.1867	−3.49	<0.001
	NO ⇒ PWB	−0.2502	0.0896	−0.4409	−0.0552	−0.1519	−2.79	0.005
	DSF ⇒ PWB	−0.0345	0.0570	−0.1605	0.0883	−0.0332	−0.61	0.545
**Total**	POSI ⇒ PWB	−0.1436	0.0960	−0.3540	0.0529	−0.0817	−1.50	0.135
	MR ⇒ PWB	−0.3772	0.0956	−0.5829	−0.1742	−0.2112	−3.94	<0.001
	NO ⇒ PWB	−0.3021	0.0888	−0.4870	−0.1146	−0.1834	−3.40	<0.001
	DSF ⇒ PWB	−0.0689	0.0569	−0.1920	0.0525	−0.0661	−1.21	0.226

*Note:* POSI—Preference for online social interaction; MR—Mood regulation; NO—Negative outcomes; DSF—Deficient self-regulation; SE—Self-esteem; PWB—Psychological well-being. Confidence intervals computed with method: Bootstrap percentiles 5000 (interval 95%); betas are completely standardized effect size.

**Table 5:** Results of the mediation analysis—Self-esteem as a mediator in the relationship between the four dimensions of general problematic internet use (preference for online social interaction, mood regulation, negative outcomes, deficient self-regulation) and social well-being (a dimension of general well-being).

Mediator Models N = 334								
Preference for online social interaction ⇒ Self-esteem ⇒ Social well-being Mood regulation ⇒ Self-esteem ⇒ Social well-being Negative outcomes ⇒ Self-esteem ⇒ Social well-being Deficient self-regulation ⇒ Self-esteem ⇒ Social well-being								
				**95% C.I.**				
**Type**	**Effect**	**Estimate**	**SE**	**Lower**	**Upper**	**β**	**z**	***p***
**Indirect**	POSI ⇒ SE ⇒ SWB	−0.0390	0.0190	−0.0901	−0.0056	−0.0245	−2.05	0.041
	MR ⇒ SE ⇒ SWB	−0.0344	0.0179	−0.0806	−0.0038	−0.0213	−1.92	0.054
	NO ⇒ SE ⇒ SWB	−0.0368	0.0190	−0.0792	−0.0028	−0.0247	−1.94	0.052
	DSF ⇒ SE ⇒ SWB	−0.0228	0.0114	−0.0541	−0.0030	−0.0243	−2.00	0.046
**Component**	POSI ⇒ SE	−0.1253	0.0404	−0.2128	−0.0386	−0.1675	−3.11	0.002
	MR ⇒ SE	−0.1224	0.0411	−0.2044	−0.0377	−0.1609	−2.98	0.003
	NO ⇒ SE	−0.1464	0.0376	−0.2197	−0.0712	−0.2086	−3.90	<0.001
	DSF ⇒ SE	−0.0829	0.0238	−0.1354	−0.0339	−0.1869	−3.48	<0.001
	SE ⇒ SWB	0.3108	0.1142	0.0888	0.5298	0.1464	2.72	0.007
**Direct**	POSI ⇒ SWB	−0.2780	0.0855	−0.4536	−0.1165	−0.1750	−3.25	0.001
	MR ⇒ SWB	−0.4329	0.0849	−0.6120	−0.2502	−0.2681	−5.10	<0.001
	NO ⇒ SWB	−0.4087	0.0790	−0.5720	−0.2426	−0.2743	−5.18	<0.001
	DSF ⇒ SWB	−0.2313	0.0501	−0.3389	−0.1241	−0.2456	−4.62	<0.001
**Total**	POSI ⇒ SWB	−0.3170	0.0853	−0.4905	−0.1566	−0.1995	−3.72	<0.001
	MR ⇒ SWB	−0.4674	0.0847	−0.6441	−0.2871	−0.2894	−5.52	<0.001
	NO ⇒ SWB	−0.4455	0.0779	−0.6049	−0.2820	−0.2990	−5.72	<0.001
	DSF ⇒ SWB	−0.2541	0.0497	−0.3610	−0.1545	−0.2699	−5.11	<0.001

*Note:* POSI—Preference for online social interaction; MR—Mood regulation; NO—Negative outcomes; DSF—Deficient self-regulation; SE—Self-esteem; SWB—Social well-being. Confidence intervals computed with method: Bootstrap percentiles 5000 (interval 95%); betas are completely standardized effect size.

## Data Availability

The data generated and/or analyzed during the current study are available from the corresponding author on reasonable request.
